# The role of CCL21/CCR7 chemokine axis in breast cancer-induced lymphangiogenesis

**DOI:** 10.1186/s12943-015-0306-4

**Published:** 2015-02-10

**Authors:** Elena Tutunea-Fatan, Mousumi Majumder, Xiping Xin, Peeyush K Lala

**Affiliations:** Department of Anatomy & Cell Biology, Schulich School of Medicine and Dentistry, Western University, 1151 Richmond St, London, ON N6A 5C1 Canada; Department of Oncology, Schulich School of Medicine and Dentistry, Western University, 790 Commissioners Rd. E, London, ON N6A 4L6 Canada; Children’s Health Research Institute, 800 Commissioners Rd. E, London, ON N6C 2V5 Canada

**Keywords:** CCL21 chemokine, CCR7 chemokine receptor, Vascular endothelial growth factor-C, Protein kinase B, Lymphangiogenesis, Breast cancer

## Abstract

**Background:**

Tumor-induced lymphangiogenesis facilitates breast cancer progression by generating new lymphatic vessels that serve as conduits for tumor dissemination to lymph nodes and beyond. Given the recent evidence suggesting the implication of C-C chemokine ligand 21/chemokine receptor 7 (CCL21/CCR7) in lymph node metastasis, the aim of our study was to define the role of this chemokine pair in breast cancer-associated lymphangiogenesis.

**Methods:**

The expression analysis of CCL21/CCR7 pair and lymphatic endothelial cell (LEC) markers in breast cancer specimens was performed by means of quantitative real-time PCR. By utilizing CCR7 and CCL21 gene manipulated breast cancer cell implants into orthotopic sites of nude mice, lymphatic vessel formation was assessed through quantitative real-time PCR, immunohistochemistry and immunofluorescence assays. Finally, the lymphangiogenic potential of CCL21/CCR7 was assessed *in vitro* with primary LECs through separate functional assays, each attempting to mimic different stages of the lymphangiogenic process.

**Results:**

We found that CCR7 mRNA expression in human breast cancer tissues positively correlates with the expression of lymphatic endothelial markers LYVE-1, podoplanin, Prox-1, and vascular endothelial growth factor-C (VEGF-C). We demonstrated that the expression of CCL21/CCR7 by breast cancer cells has the ability to promote tumor-induced lymph-vascular recruitment *in vivo. In vitro*, CCL21/CCR7 chemokine axis regulates the expression and secretion of lymphangiogenic factor VEGF-C and thereby promotes proliferation, migration, as well as tube formation of the primary human LECs. Finally, we showed that protein kinase B (AKT) signaling pathway is the intracellular mechanism of CCR7-mediated VEGF-C secretion by human breast cancer cells.

**Conclusions:**

These results reveal that CCR7 and VEGF-C display a significant crosstalk and suggest a novel role of the CCL21/CCR7 chemokine axis in the promotion of breast cancer-induced lymphangiogenesis.

**Electronic supplementary material:**

The online version of this article (doi:10.1186/s12943-015-0306-4) contains supplementary material, which is available to authorized users.

## Background

Chemokines and their receptors play essential roles in tumor biology including leukocyte recruitment, tumor cell growth and survival, angiogenesis, and metastasis [1-7]. Among them, C-C chemokine ligand 21/chemokine receptor 7 (CCL21/CCR7) pair promotes growth and metastasis of many tumor types including melanomas, breast, thyroid, colon, head, and neck cancers [8-14]. CCR7 has emerged as an important marker in the prediction of axillary lymph node metastasis in breast carcinomas, particularly since CCR7 over-expression correlates with larger primary tumors, deeper lymphatic invasion and poorer survival rates [10,13,15]. *In vivo* studies revealed that metastatic tumor formation is decreased when CCL21 expression is knocked down in secondary lymphoid organs, since this diminishes both the chemotactic and antiapoptotic effects of CCR7-expressing tumor cells [14]. Similarly, CCL21/CCR7 pair seems to play an important role in the lymphangiogenesis associated with pancreatic cancer [16,17] and – due to its chemotactic properties – this chemokine axis is involved in the lymphatic spread of melanoma cells [18]. However, while the complete picture on the role and involvement of CCL21/CCR7 pair in breast cancer is still undergoing development, there are at least two areas in which this axis has shown to be actively involved, often through vascular endothelial growth factor C (VEGF-C) mediated signaling, namely: lymph nodes metastasis, and immune response modulation [18-23].

VEGF-C production by tumor cells is recognized as the chief promoter of tumor-associated lymphangiogenesis by stimulating growth and differentiation of lymphatic endothelial cell precursors [24-26]. Tumor-derived VEGF-C can also mediate lymphangiogenesis-independent actions that promote breast cancer invasiveness and metastasis [27,28]. We had earlier reported that overexpression of cyclooxygenase-2 (COX-2) in breast cancer cells – resulting in increased prostaglandin E2 (PGE2) levels in the tumor milieu – promotes metastasis by multiple mechanisms including stimulation of tumor cell migration [29,30], invasiveness [31], tumor-associated angiogenesis [29], and lymphangiogenesis [32-34] caused by an upregulation of VEGF-C secretion via prostaglandin EP1/EP4 receptors [27,32,33]. Along the same lines, EP2 receptor has been shown to be involved in COX-2 mediated lymphangiogenesis [35]. However, neither COX-2 inhibitors nor EP4 antagonists could completely abrogate VEGF-C production by highly metastatic breast cancer cells indicating that additional mechanisms are involved in VEGF-C secretion. While prior studies have established that COX-2 secretion by breast cancer cells can upregulate CCR7 expression via activation of EP2/EP4 receptors [20,36] to enhance their invasive capacity, a possible link between CCR7 signaling and VEGF-C expression/secretion has remained untested so far. Therefore, the objective of the present study was to investigate whether CCL21/CCR7 signaling promotes breast cancer-associated lymphangiogenesis through CCR7-dependent stimulation of VEGF-C secretion followed by LECs activation towards the development of new lymphatic vessels. This objective was achieved by a combination of *in situ, in vivo,* and *in vitro* approaches.

Here, we have established that CCR7 correlates with the expression of lymphatic endothelial cell markers in a panel of human breast cancer tissues as well as with the expression of the lymphangiogenic factor VEGF-C. By utilizing CCR7 or CCL21 gene manipulated breast cancer cell implants *in vivo* we have shown that the analyzed chemokine pair promotes host lymphatic vessel recruitment and growth. Moreover, CCL21/CCR7 chemokine axis has the ability to promote lymphatic endothelial cells proliferation, migration, as well as tube formation *in vitro,* and this axis also regulates the expression of lymphangiogenic factor VEGF-C by breast cancer cells. Finally, the phosphorylation of AKT pathway constitutes the intracellular mechanism underlying CCR7-mediated VEGF-C synthesis. Our study adds new elements to the multifaceted role of CCL21/CCR7 chemokine pair in mammary malignancy by revealing a novel role of this chemokine axis in breast cancer-associated lymphangiogenesis that might be relevant to future therapies.

## Results

### Role of CCL21/CCR7 pair in mediation of VEGF-C secretion by breast cancer cells

**Figure 1:**
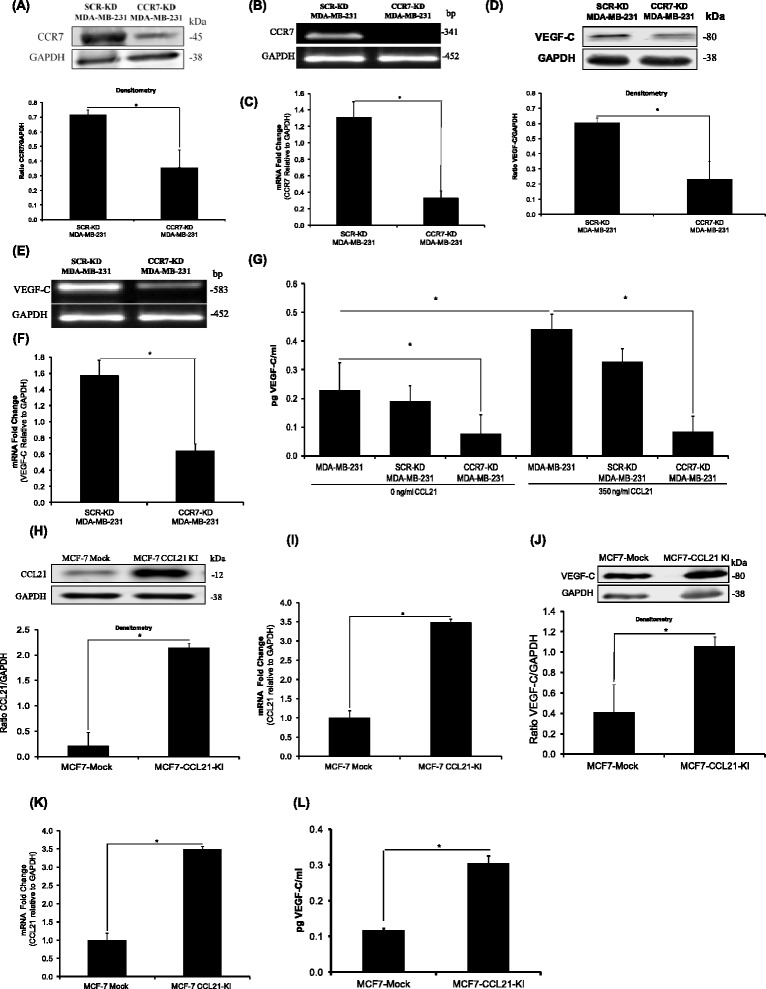
**CCR7 promotes expression and secretion of lymphangiogenic factor VEGF-C. (A)** Western blots **(B)** real-time PCR and **(C)** quantitative real-time PCR validating CCR7 knockdown in MDA-MB-231 cells versus control shRNA. **(D)** Western blot, **(E)** real time PCR and **(F)** quantitative real-time PCR analysis outlining decreased VEGF-C expression in MDA-MB-231 CCR7 shRNA cells versus control shRNA. **(G)** ELISA demonstrates that VEGF-C protein concentration in conditioned media from CCL21 treated/untreated CCR7-KD cells decreased significantly compared to parental or scrambled MDA-MB-231 cells. **(H)** Western blot and **(I)** quantitative real-time PCR validating CCL21 overexpression in MCF-7 cells. **(J)** Western blot, **(K)** quantitative PCR outlining increased VEGF-C expression in MCF-7 CCL21 cells versus control mock. **(L)** VEGF-C protein concentration in conditioned media from MCF-7 CCL21 and control cells as measured by ELISA. In **(A-K)** data are represented as mean ± SD (n = 4), except **(G)** and **(L)** where data are represented as mean ± SD (n = 3). (*) indicates statistical significant differences (p < 0.005).

Prior to the investigation of the role of CCL21/CCR7 pair in VEGF-C production, we have screened the constitutive expression of CCR7, CCL21, and VEGF-C in two well differentiated, luminal type (T47D, MCF-7) and two poorly differentiated basal type (Hs578t, MDA-MB-231) breast cancer cell lines (Additional file 1: Figure S1A and B). Based on these preliminary results, MDA-MB-231 breast carcinoma cell line – that is characterized by an invasive phenotype – was selected for its ability to express/secrete high levels of VEGF-C, which makes it adequate for use in a loss-of-function model. Conversely, for the gain-of-function approach, MCF-7 cell line was selected since expresses/secretes relatively low levels of VEGF-C. In this regard, CCR7 expression in MDA-MB-231 cells was knocked down with shRNA targeting CCR7 gene and the effectiveness of transfection was assessed by means of Western blot, real-time PCR, and quantitative real-time PCR (Figure 1A to C). Of note, low levels of CCR7 expression correlates with significant downregulations in VEGF-C protein and mRNA expressions (Figure 1D to F). To determine whether CCL21/CCR7 interaction regulates the secretion of lymphangiogenic factor VEGF-C, CCR7 shRNA and parental MDA-MB-231 cells were incubated with and without human CCL21/6Ckine (350 ng/ml) for 24 hours. While exogenous CCL21 has stimulated VEGF-C production by parental cells, the secretion level of VEGF-C from CCR7 shRNA transfected tumor cells has decreased significantly. The level was significantly lower compared to CCL21 treated or untreated parental cells or scrambled shRNA transfected cells (Figure 1G). Moreover, the proposed molecular mechanism responsible for the regulation of VEGF-C was analyzed in MCF-7 mock and MCF-7-CCL21-knocked in (KI) breast cancer cells and the efficacy of nucleotransfection is presented in Figure 1H and I. Quantitative PCR, Western blot, and ELISA confirmed that CCL21/CCR7 pair has the ability to regulate the expression of the lymphangiogenic factor VEGF-C in the analyzed MCF-7 breast cancer cells (Figure 1H - L). Overall, these findings suggest that CCL21/CCR7 pair has the potential to regulate VEGF-C expression/secretion in the analyzed breast cancer cell line models.

### Signaling mechanisms of CCR7-mediated VEGF-C secretion

Prior studies have documented the role of phosphatidylinositol 3-kinase (PI3K) and its downstream mediator protein kinase B (AKT) in the survival and invasiveness of head and neck carcinoma cells through CCL21/CCR7 interaction [37,38]. Furthermore, extracellular signal-regulated kinase (ERK1/2) pathway is involved in cell cycle progression and survival of non-small cell lung carcinoma cells resulting from CCR7 activation [39,40].

**Figure 2:**
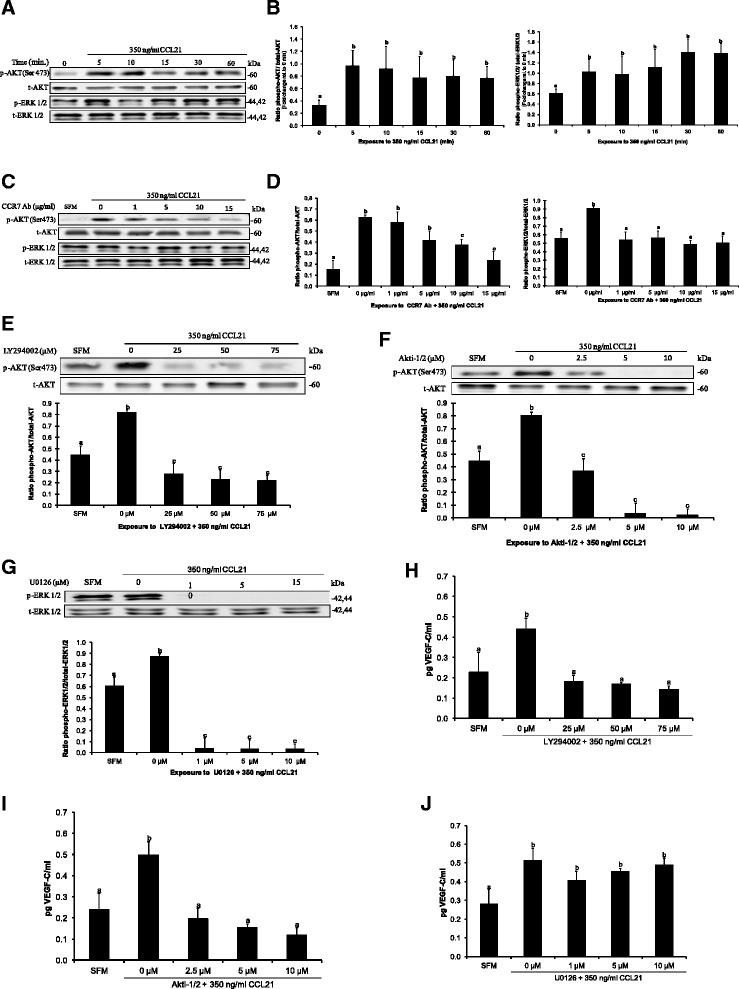
**CCR7 activation by CCL21 regulates VEGF-C secretion via the PI3K/AKT signaling pathway. (A)** Western blot time course analysis of AKT and ERK1/2 activation in MDA-MB-231 cells following treatment with CCL21 for a total of 60 minutes. Phosphorylation of AKT at Ser 473 and ERK1/2 is observed over the entire duration of stimulation compared to control, untreated cells. **(B)** Densitometric analysis revealed that phosphorylation of both pathways is significantly induced after 5 min of CCL21 treatment. **(C)** Western blot and **(D)** Densitometric analysis indicating that CCL21-induced phosphorylation is dependent on CCR7 activation. Western blot demonstrating that PI3K/AKT inhibitors LY 294002 **(E)** Akt-1/2 **(F)**, and ERK1/2 inhibitor U0126 **(G)** can block CCL21/CCR7-mediated phosphorylation in an inhibitor dose-dependent manner. For all experiments, total AKT and total ERK1/2 confirmed the equivalent loading of lanes. **(H-J)** PI3K/AKT but not ERK1/2 specific inhibitors significantly block CCL21 mediated VEGF-C secretion measured with ELISA. Data are represented as means ± SD (n = 3). Different superscripts indicate statistically significant differences (p < 0.05).

To investigate whether AKT and ERK1/2 pathways are downstream activated by CCL21-CCR7 binding, the phosphorylation status of both pathways was assessed in MDA-MB-231 breast cancer cells. As expected, an increase in phosphorylation of AKT and ERK1/2 was observed for the entire duration of stimulation with CCL21/6Ckine (Figure 2A, B). To assess whether the phosphorylation of both proteins was dependent on the activation of CCR7, MDA-MB-231 cells were treated with various concentrations of CCR7 antibody (0, 5, 10, 20 μg/ml) before stimulation with CCL21/6Ckine. When CCR7 was blocked by its neutralizing antibody, the phosphorylation status of AKT and ERK1/2 was significantly decreased (Figure 2C, D). Similarly, the addition of PI3K/AKT and ERK1/2 specific inhibitors significantly reduced the effect of CCL21 on activation of both pathways (Figure 2E - G).

Finally, to test the functional roles of PI3K/AKT and/or ERK1/2 pathways in CCL21-induced VEGF-C secretion, MDA-MB-231 cells were pre-treated with various concentrations of PI3K inhibitor (LY294002 at 25, 50, and 75 μM), Akt-1/2 inhibitor (at 2.5, 5, and 10 μM), and ERK1/2 inhibitor (U0126 at 5, 10, and 15 μM) and then treated with CCL21/6Ckine (350 ng/ml) for 24 hours. Following that, the supernatants were collected and subjected to a VEGF-C ELISA. Both PI3K and AKT inhibitors yielded a significantly reduced VEGF-C secretion when compared with CCL21 treatment alone (Figure 2H, I), whereas no significant difference was observed for U0126 treatment (Figure 2J). As such, it can be affirmed that the activation of PI3K/AKT, but not ERK1/2 signaling pathway downstream of CCR7 is involved in the regulation of VEGF-C secretion.

### CCL21/CCR7 pair has lymphangiogenic potential *in vitro*

**Figure 3:**
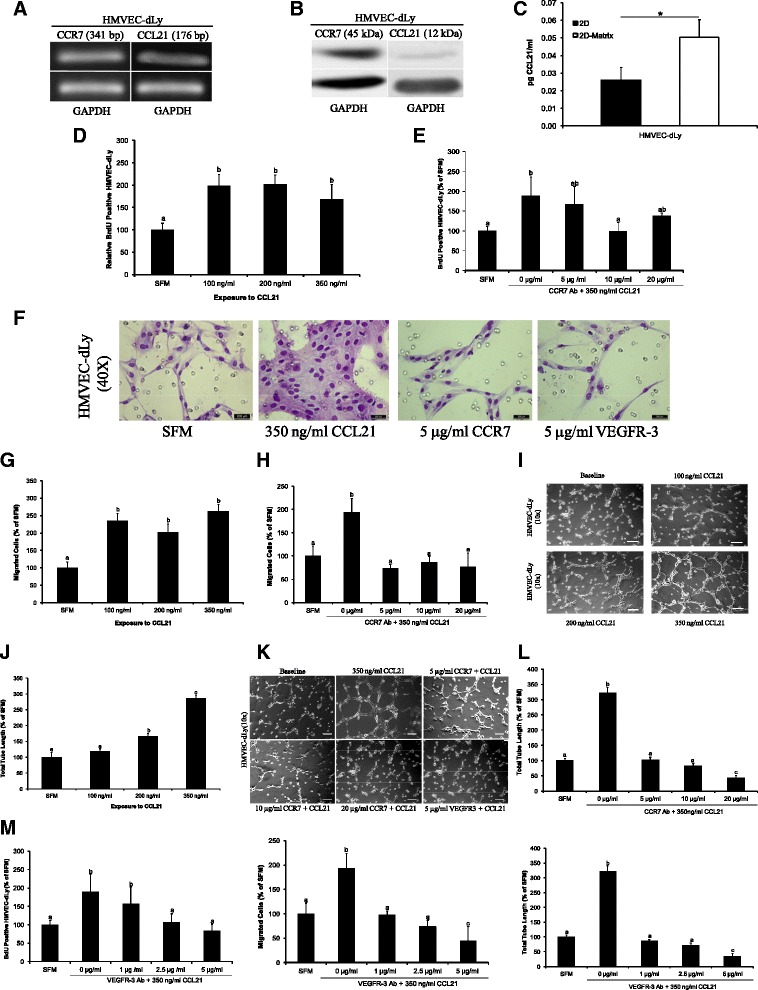
**CCL21/CCR7 axis has lymphangiogenic potential** ***in vitro.*** **(A)** Real-time PCR of CCR7 and CCL21 mRNA expression in HMVEC-dLy. **(B)** Western blot of CCR7 and CCL21 protein expression in HMVEC-dLy. GAPDH was used as an internal control. **(C)** CCL21 protein secretion by HMVEC-dLy as measured by ELISA. Data are represented as mean ± SD (n = 3). (*) indicates significant difference (p < 0.05). **(D)** HMVEC-dLy proliferation in response to CCL21/6Ckine (0, 100, 200, 350 ng/ml) performed by the measurement of BrdU. Data are presented as mean ± SD (n = 4, p < 0.001). **(E)** HMVEC-dLy proliferation in response to CCR7 neutralizing antibody (0, 5, 10, 20 μg/ml). Data are presented as mean ± SD (n = 4, p < 0.0003). **(F)** Representative images of HMVEC-dLy migration (40× magnification). **(G)** Quantification of HMVEC-dLy cellular migration in response to CCL21/6Ckine (0, 100, 200, and 350 ng/ml). **(H)** Quantification of HMVEC-dLy migration in response to CCR7 neutralizing antibody (0, 5, 10, 20 μg/ml). Bars in **(G, H)** represent mean number of migrated cells ± SD (n = 4, p < 0.005). **(I)** Representative micrographs of HMVEC-dLy tubular network formation in response to CCL21/6Ckine (0, 100, 200, 350 ng/ml). Bar equals 100 μm. **(J)** Quantification of total length of tubular structures formed by HMVEC-dLy corresponding to **(I)** as determined by ImageJ. **(K)** Representative images of inhibition of HMVEC-dLy tubular network formation. Bar equals 100 μm. **(L)** Quantified HMVEC-dLy tube formation corresponding to images shown in **(K)**. In **(J and** **L)** data are presented as mean ± SD (n = 4, p < 0.0001). **(M)** Quantified HMVEC-dly proliferation, migration, and tube formation in response to treatment with VEGFR-3 neutralizing antibody (0, 1, 2.5, 5 μg/ml). Data are presented as mean ± SD. Different superscripts represent a statistical significant difference.

Lymphatic vessel formation is a complex biological process that can be approached *in vitro* through separate assays, each attempting to mimic a different stage of the lymphangiogenic process such as: proliferation, migration, and formation of capillary-like tubes [41]. Within the limited scope of the current study, primary HMVECs-dLy were employed as an *in vitro* model of LECs and their CCR7 and CCL21 expressions were verified at the mRNA and protein levels. Agarose gel electrophoresis of real-time PCR products from HMVEC-dLy cells showed that both the chemokine receptor and ligand are expressed at the mRNA level (Figure 3A). On the other hand, lysates from cultured HMVEC-dLy cells were assayed by Western blot and CCR7 protein expression was detected at 45 kDa while only traces of CCL21 were observed (Figure 3B). Moreover, CCL21 protein secretion by HMVEC-dLy cells was quantified in 2D and 2D-matrix conditions (Matrigel was used because CCL21 is strongly matrix-binding). One important aspect to be emphasized is that CCL21 chemokine ligand is secreted as a small molecular weight protein that is readily immobilized within the extracellular matrix by binding to sulfated proteoglycans [42]. As such, there was no surprise that the determined bound CCL21 protein fraction was about twofold higher than the soluble fraction, but the overall CCL21 secretion by HMVEC-dLy was constitutively present at very low levels (Figure 3C). Therefore, serum-starved LECs were treated with various concentrations of exogenous CCL21/6Ckine (100–350 ng/ml) and their proliferation rates (BrdU uptake) increased significantly (Figure 3D). Conversely, when CCR7 activation was blocked with CCR7-neutralizing antibody (10 μg/ml), LECs proliferation significantly decreased to serum-free level (Figure 3E). LECs also responded to the chemotactic effect of CCL21 by increased migration (Figure 3F, G). Conversely, in the presence of various concentrations of CCR7-neutralizing antibody (5, 10, 20 μg/ml), CCL21-mediated LECs migration was significantly inhibited (Figure 3H). Finally, the extent of tube-like structures formed by LECs increased significantly with increasing concentrations of CCL21/6Ckine (100–350 ng/ml) (Figure 3I, J). By contrast, CCL21 mediated tubulogenesis was blocked by CCR7 neutralizing antibody (Figure 3K, L). To determine whether CCL21/CCR7 acts through the regulation of VEGFR-3 ligand, LECs were treated with CCL21 in the presence and absence of VEGFR-3 neutralizing antibody (1, 2.5, 5 μg/ml). Blocking VEGFR-3 significantly has reduced CCL21-induced LECs proliferation, migration, and tube formation (Figure 3M). Based on these results, it can be inferred that within the framework of the investigated *in vitro* model, CCL21/CCR7 pair has a direct lymphangiogenic potential through stimulation of the lymphangiogenic traits of LECs. Additionally, CCL21/CCR7 pair interacts in a ligand-independent manner with VEGFR-3 expressed by LECs to drive their lymphangiogenic response.

### CCL21/CCR7 pair promotes lymph-vascular recruitment *in vivo*

**Figure 4:**
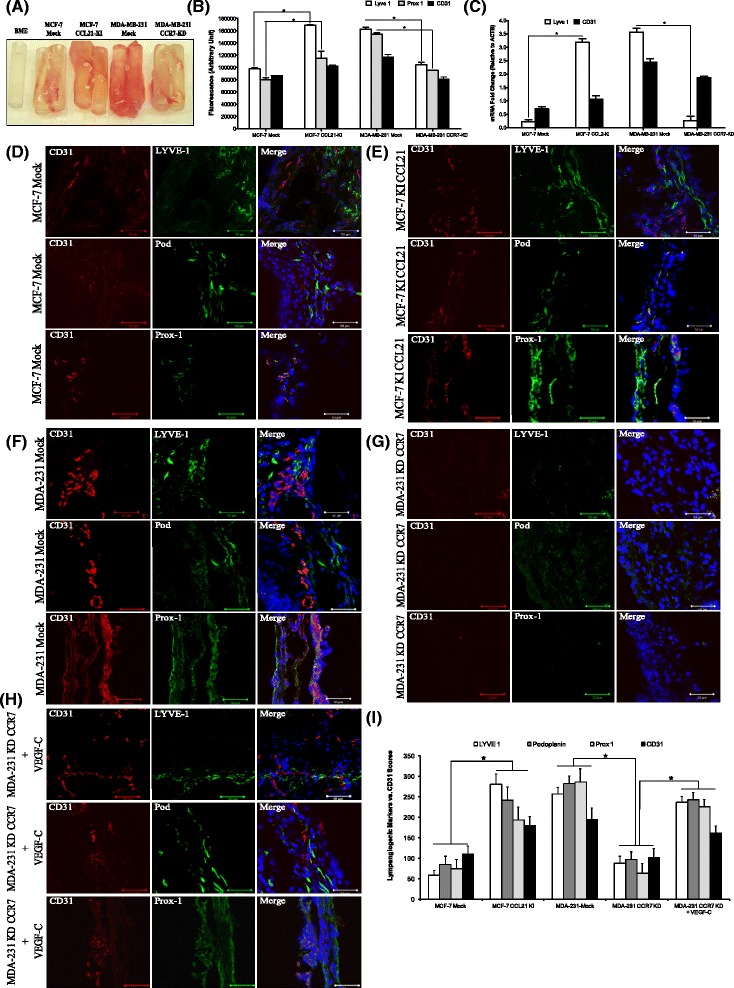
**CCL21/CCR7 axis has lymphangiogenic potential** ***in vivo*** **. (A)** Macroscopic digital images of angioreactors collected with surrounding tissues. **(B)** Fluorescence assay analysis of LYVE1, Prox1, and CD31 markers as expressed by mouse lymphatic endothelial cells and blood vascular endothelial cells recruited into the angioreactors. Data are presented as mean relative fluorescent units ± SEM. (*) indicates significant difference (p < 0.005). **(C)** Quantitative real-time PCR analysis of LYVE1 and CD31 mRNA expression in the cellular contents of angioreactors. Expression levels are normalized to actin (ACTB). (*) indicates significant difference (p < 0.05) (n = 4). **(D to G)**. Representative images of immunofluorescence localization of CD31 (red), LYVE1 (green), Podoplanin (green), and Prox1 (green) in serial sections of angioreactors containing MCF-7 mock vs. CCL21 KI MCF-7 cells **(D)**, **(E)** and MDA-MB-231 mock vs. CCR7 KD MDA-MB-231 cells **(F)**, **(G)**. Nuclei are stained with DAPI (blue). Scale bar equals 50 μm. **(H)** Representative images of immunofluorescence localization of CD31 (red), LYVE1 (green), Podoplanin (green), and Prox1 (green) in serial sections of angioreactors containing CCR7 KD MDA-MB-231 cells and recombinant human VEGF-C (30 ng/μl). Nuclei are stained with DAPI (blue). Scale bar equals 50 μm. **(I)** Quantification of MVD and LVD. “Hot spot” scores for CD31-LYVE1, CD31-Podoplanin, and CD31-Prox1 were calculated by means of Image J (40× magnification). Data are presented as mean of “hot spot” ± SEM.

To validate our *in vitro* observations, Directed *In Vivo* Lymphangiogenic Assay (DIVLA) was conducted, as reported in the past [43]. In brief, the angioreactors containing basement membrane extract and breast cancer cells were implanted subcutaneously into the dorsal flanks of nude mice and lymphatic vessel formation was assessed through multiple approaches. Host lymphatic endothelial cells that invaded the angioreactors associated with different conditions were quantified with an immunofluorescence assay for LYVE1 and Prox1. The results revealed that the recruitment of LYVE1 and Prox1 labelled lymphatic endothelial cells was significantly higher in the angioreactors containing MCF-7-CCL21-KI cells when compared with their controls. Similarly, this recruitment was impaired when CCR7 was knocked down, as observed in the MDA-MB-231-CCR7-KD angioreactors compared to control, MDA-MB-231-mock angioreactors (Figure 4B). Relative LECs recruitment measured with quantitative real-time PCR for LYVE1 mRNA expression in cellular contents of the angioreactors also revealed a similar phenomenon: a significantly higher recruitment with MCF-7-CCL21-KI than with MCF-7-mock cells; and a significantly lower recruitment with MDA-MB-231-CCR7-KD than with MDA-MB-231 mock cells (Figure 4C). For a direct quantification of MVD and LVD, serial cryosections of angioreactors were performed and dual immunostaining was subsequently used to visualize lymphatics (LYVE1/Prox1/Podoplanin) and blood vessels (CD31) by means of “hot spot” method [34,43]. Higher MVD and LVD were observed in MCF-7-CCL21-KI sections when compared to control (MCF-7 mock). Similarly, lower densities were observed in MDA-MB-231-CCR7-KD sections than in MDA-MB-231 mock (Figure 4D – G, quantification 4I). Finally, the addition of recombinant VEGF-C to the angioreactors containing MDA-MB-231-CCR7-KD cells has rescued lymphatic vessel formation to the same level observed with control MDA-MB-231 (Figure 4H). To verify the *in vivo* expression of CCR7 by LECs, angioreactors containing only lymphangiogenic growth factors were implanted into nude mice and quantitative real-time PCR analysis was performed to analyze the content of the tubes (Additional file 2: Figure S2B). Thus, according to our *in vivo* results, CCL21/CCR7 pair is a positive regulator of lymphangiogenesis.

### CCR7 expression correlates with lymphangiogenic markers in breast cancer samples

**Figure 5:**
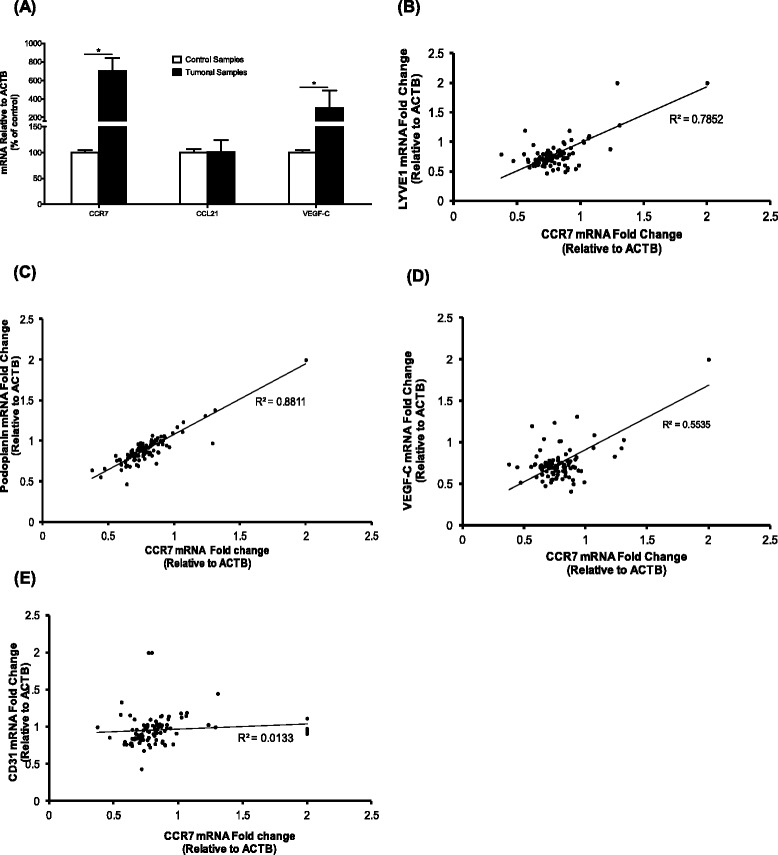
**CCL21/CCR7 pair correlates with lymphangiogenic markers in a panel of breast cancer tissues. (A)** Quantitative real-time PCR analysis of CCL21, CCR7 and VEGF-C mRNA expression in control (adjacent non-tumor) and tumoral tissues. Data are represented as a mean ± SD. (*) indicates significant differences (p < 0.05). **(B)**, **(C)**, **(D)** mRNA expression level of CCR7 is positively correlated with the expression of lymphatic vascular markers (LYVE1, Podoplanin, VEGF-C) in primary breast cancer samples; Pearson’s coefficient indicates strong correlations. **(E)** mRNA expression level of CCR7 and blood vessel marker CD31 in primary breast cancer samples. Pearson’s coefficient suggests little to no correlation between the two variables. Data represent mean values from three independent experiments.

To characterize the role of CCL21/CCR7 *in situ*, we used a panel of breast cancer tissues (n = 105) collected from the primary tumor site. The demographic and clinical characterization of the samples is summarized in Additional file 3: Table S1. In brief, the majority of the tissues were classified as invasive mammary carcinomas characterized by various grades of differentiations: grade III (62%) and grade II (24%). At first, quantitative real-time PCR was employed to compare the expression profile of CCR7, CCL21, and VEGF-C in primary tumor and adjacent breast tissues (control). The stability in expression levels of two common housekeeping genes (GAPDH and ACTB) was verified across the entire panel of tissues. Of note, the level of CCR7 mRNA in tumor tissues was significantly higher than that in the control samples. A trend similar to CCR7 was observed for VEGF-C. However, CCL21 mRNA expression in tumors was not significantly different when compared to controls (Figure 5A). Moreover, it was found that CCR7 mRNA expression is positively correlated with lymph-vascular markers (LYVE1, podoplanin) and VEGF-C in breast cancer specimens while no correlation with endothelial marker CD31 was observed (Figure 5B – E). As for CCL21 expression, a weak correlation with lymph-vascular markers was observed (data not included). These results suggest a clinical association between CCR7 expression and lymph-vascular recruitment in primary breast tumor.

## Discussion

Lymphangiogenesis, the growth of new lymphatic vessels, has an important role in the complex pathology of tumoral processes in general, and in that of breast cancer in particular [24,25,44,45]. Furthermore, lymphatics have started to be reclassified as active, rather than passive conduits in cancer, since they are able to fine tune the balance between peripheral tolerance and immunity that facilitates host immune tolerance to tumor invasion [46,47]. The newly cited roles of lymphatics include the indirect suppression of T-cell function, the inhibition of dendritic cell (DC) maturation, as well as a direct effect on T-cell tolerance [48].

Within the immunological context, the two major leukocyte subsets whose surfaces express CCR7 are dendritic and T cells such that CCR7 is known to have strong implications on both central and peripheral tolerance [49]. On the other hand, CCL21 is constitutively expressed by the lymphatic endothelium of multiple organs, high endothelial venules of lymph nodes and Peyer’s patches, as well as stromal cells in T cell rich areas of lymph nodes, and spleen [50]. The wide physiological distribution, combined with the complex and multifaceted roles in lymph node trafficking could be one of the reasons for which CCL21/CCR7 axis has been identified as a viable candidate for the fast dissemination of breast cancer cells developed in the immediate proximity of the lymphatics. Furthermore, the lymphangiogenic factor VEGF-C is one of the known promoters of intra- and peritumoral lymphatic development and a facilitator of tumor cell dissemination [26,33,51]. However, molecular regulation of lymphangiogenesis extends to many other types of interactions that are often placed outside of the conventional VEGF family of pro-lymphangiogenic factors [17,52-54].

**Figure 6:**
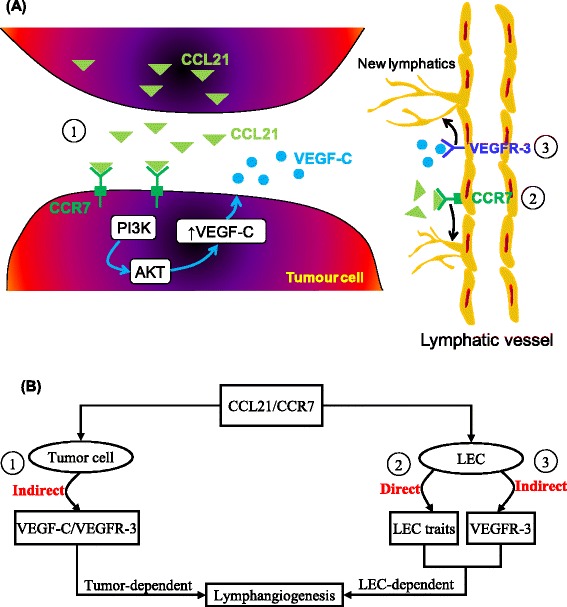
**Schematic diagram of the proposed role of CCL21/CCR7 axis in lymphangiogenesis. (A, B)** Molecular crosstalk between tumor cells and LECs. 1) Tumor cells enhance the expression of VEGF-C in response to CCL21/CCR7 signaling and promote sprouting of new lymphatics. 2) CCR7-expressing LECs have a direct lymphangiogenic potential by stimulating the pro-lymphangiogenic traits of LECs. 3) LECs respond to CCL21/CCR7 axis through the modulation of VEGFR-3 signaling pathway.

The involvement of CCL21/CCR7 pair in migration and guidance of the cells detached from the primary tumor towards draining lymphatics is believed to play a significant role in the subsequent metastatic evolution of the disease [18,19]. Present results, combined with our earlier report of pro-migratory functions of VEGF-C [27], and reported VEGF-C mediated stimulation of CCL21 secretion by LECs [21], endorses the idea that the interplay/crosstalk between CCL21 chemokine and VEGF-C promotes breast cancer progression through several distinct, but complementary mechanisms. Our present results prompt for the first time that a closed loop/circular communication exists between CCL21/CCR7 and VEGF-C/VEGFR-3 axes, in a sense that not only VEGF-C promotes CCL21 secretion by LECs [21], but also that CCR7 activation stimulates VEGF-C synthesis by tumor cells and thus drives lymphangiogenesis. Indeed, our results show that inhibition of CCR7 gene translates into significant decreases of VEGF-C expression in the analyzed breast cancer cells. In addition, our study reports a significant inducible VEGF-C secretion in MDA-MB-231 cells in response to CCR7 activation (Figures 1, 2, and 4). Moreover, CCL21 has been found to regulate lymphangiogenesis in a LEC-dependent manner (Figure 3): 1) directly, through the stimulation of the lymphangiogenic traits of LECs (*e.g.* proliferation, migration and tubular network formation); and 2) indirectly, through the modulation of VEGFR-3 signaling pathway. An overall summary of these findings is depicted in the schematic of Figure 6. Furthermore, these results corroborate well with our *in situ* findings outlining a strong correlation between CCR7 and VEGF-C expression in human breast cancer tissues. However, CCR7 may activate other lymphangiogenic factors to mediate lymphatic vascularisation, such that a full analysis of its pro-lymphangiogenic profile/signature represents an avenue that to be explored in the future.

Previous studies indicate that CCR7-mediated signaling required for invasive and pro-survival functions in head and neck cancer cells is mediated by PI3K/AKT pathway [14], whereas CCR7-mediated cell cycle progression in lung cancer cells utilizes ERK1/2 pathway [39]. We demonstrate here that CCR7 activation by CCL21 binding can induce a significant increase in both AKT and ERK1/2 phosphorylation in breast cancer cells; however, only the former pathway is required for VEGF-C up-regulation and secretion. While this result constitutes a new finding in the context of CCR7 chemokine receptor, a similar molecular mechanism was confirmed for insulin-like growth factor-I receptor-mediated VEGF-C secretion in lung carcinoma cells [55].

Another important finding in our study is that CCL21/CCR7 axis has a significant effect on LECs proliferation, migration, and tubular network formation and this effect seems to be regulated by means of VEGFR-3 signaling pathway. However, the precise molecular mechanisms involved in LEC-dependent/tumor-independent lymphangiogenesis remain to be elucidated. Interestingly, neither CCL21 stimulation nor CCR7 receptor blocking induced dose-dependent effects on HMVEC-dLy proliferation and migration, a phenomenon that could be explained perhaps through chemokine receptor saturation followed by its desensitization and internalization [56]. Another important aspect to be emphasized here is that by contrast with the surveyed literature [57,58] reporting that lymphatic cells are capable of secreting CCL21, HMVEC-dLy cells used in the present study were found as weak producers of CCL21 chemokine. One possible explanation of this result could be the substantial changes in gene expression induced by culture in primary cells that might be the cause of the loss of CCL21 production [59]. Cell culture might therefore alter some of the core features of HMVEC-dLy cells.

Our *in vivo* findings indicate that CCR7 is expressed by LECs and therefore promotes LECs recruitment and lymphangiogenesis through the regulation of the expression/secretion of VEGF-C by human breast cancer cells. To recapitulate, we have created a directed *in vivo* lymphangiogenic assay (DIVLA) wherein in as a loss-of-function model the CCR7 expression was knockdown and a significant decrease in lymphatic vessel density was remarked, a phenomenon that was rescued by the addition of lymphangiogenic factor VEGF-C. These results reiterate the effects which we have observed in our comparable gain-of-function model, in which an increase in CCL21 expression associates well with an increase in lymphatic vessel density.

Since our *in vitro* and *in vivo* studies have established the roles of CCR7/CCL21 and VEGFR3/VEGF-C axes in breast cancer induced lymphangiogenesis, we have postulated that CCR7 and VEGF-C expressions will be noticeably different in tumor tissues when compared to their non-tumor counterparts. Herein our study provides evidence that the expressions of both CCR7 and VEGF-C were significantly upregulated in the analyzed breast carcinoma samples when compared with control non-tumor tissues. Interestingly, no significant difference in CCL21 expression was noted between tumor and non-tumor regions. Similar variation patterns were also observed for CCR7 and CCL21 in pancreatic cancer [16], for CCL21 in squamous cell carcinoma [60] or for CCR7 and VEGF-C in pancreatic ductal adenocarcinoma [17]. In addition, we have identified positive correlations between the CCR7 expression and lymphatic endothelial markers in the analyzed panel of breast cancer tissues.

## Conclusions

Collectively, these results add newer insights into the multifaceted role played by the CCL21/CCR7 chemokine pair in mammary malignancy, prompting for the first time towards the involvement of this chemokine axis in the complex mechanics of breast cancer-induced lymphangiogenesis The proven therapeutic effectiveness of blocking CCR7-mediated CCL21 signaling [61] combined with the promising outlook [62] of phase 1 DC-CCL21 trial in lung cancer and melanoma patients [63], suggests that the inherent value of the present study is more than apparent.

## Methods

### Ethics statements

Human breast cancer specimens and the adjacent non-tumor tissues used in this study were obtained from the Ontario Institute for Cancer Research (OICR) repository (Ontario Tumor Bank) following approval by their Ethics Board. The experimental protocol involving animals was approved by the Animal Use Subcommittee of Western University, according to the guidelines of the Canadian Council on Animal Care.

### Human tissue samples

Frozen human breast cancer tissues (n = 105) were collected from the primary tumor site with a majority being classified as invasive mammary carcinomas. Control tissues (n = 20) were obtained from adjacent non-tumor tissue from unrelated patients and subjected to histopathological analysis to confirm their status. Analyzed samples included variable amounts of duct, stroma and adipose tissue. A summary of demographic, estrogen receptor (ER), progesterone receptor (PR), and human epidermal growth factor receptor-2 (HER-2) status for patient and control populations is presented in Additional file 3: Table S1. The majority of the cancer patients (>80%, data not provided) had a history of some unspecified cancer in the family. Of the tumor tissues, 76% were ER positive, 62.9% PR positive, 20% HER2 positive, and 9.5% triple (ER/PR/HER2) negative.

### Cell lines and culture

Primary Adult Human Dermal Lymphatic Microvascular Endothelial Cells (HMVEC-dLyAd) were obtained from Clonetics®/Lonza (Walkersville, MD, USA). The initial expansion and subsequent passages (maximum of 5) were performed according to the manufacturer’s instructions. Human MDA-MB-231 and MCF-7 breast cancer cell lines from American Type Culture Collection (ATCC) (Rockville, MD, USA) were grown in a humidified incubator at 37°C with 5% CO_2_ and maintained as per instructions.

### Gene knockdown/knockin and stable transfection

MDA-MB-231 cells (10^6^ cells/ml), were transfected with either silencer small hairpin interfering RNA (shRNA) targeting CCR7 (Gene ID: 1236, TF314124, OriGene Technologies, Rockville, MD, USA) or silencer negative control shRNA (TR30015, OriGene Technologies) constructs using the Amaxa Cell Line Nucleofector Kit (Lonza, Walkersville, MD, USA). MCF-7 cells (3×10^6^ cells/ml) were transfected with either GFP-tagged ORF clone of CCL21 (Gene ID: NM_002989, RC206579, OriGene Technologies) or control plasmid vector (pCMV6-C) (PS100010, OriGene Technologies). For stable selection, Puromycin (300 ng/ml) and Geneticin (400 ng/ml) were used on MDA-MB-231 and MCF7 cells, respectively. The nucleofection efficiency was determined with quantitative real-time PCR and Western blot.

### RNA extraction, cDNA synthesis, and reverse transcriptase polymerase chain reaction (RT-PCR)

Total RNA was extracted using the RNeasy Minikit (Qiagen, Valencia, MD, USA) following manufacturer’s instructions. cDNA was synthesized with a High Capacity cDNA Reverse Transcription kit (Applied Biosystems, Carlsbad, CA, USA) using up to 2 μg of RNA. Primers for the human CCR7, CCL21, VEGF-C, and housekeeping gene (GAPDH) were synthesised at the Oligo Factory (London, ON, Canada) (Additional file 4: Table S2). PCR was performed with Platinum PCR SuperMix High Fidelity (Invitrogen, Burlington, ON, Canada). Real-time PCR products were visualized by GelRed Nucleic Acid Gel Stain (Biotium, Hayward, CA, USA) using a gel imaging system (Gel Doc™ XR System, Bio-Rad, Mississauga, ON, Canada).

### RNA extraction from human breast tissue

In order to obtain optimal RNA yield and purity, tissues were initially cut with sterile surgical blades to remove the surrounding fat. Following fat removal, the product was weighted to not exceed 30 mg and further subjected to disruption and uniformization with rotor-stator homogenizer (flash sonication for 5 seconds × 5 times) to ensure the appropriate release of RNA as well as the reduction of lysate viscosity. Then, samples were subjected to RNeasy Minikit protocol (Qiagen).

### Quantitative real-time PCR

Reaction was performed in single micro capillary tubes on a LightCycler (Roche Diagnostic, Laval, QC, Canada) with TaqMan® Universal PCR Master Mix (Applied Biosystems, Foster City, CA, USA) for control and target gene expression primer probes (TaqMan® Gene Expression Assay, Applied Biosystems) (Additional file 5: Table S3). Delta-delta Ct (ΔΔCt) method was employed to determine the fold difference (2 ^–ΔΔCt^) (Applied Biosystems).

### Western blot

To prepare protein lysates, cells were treated with M-PER® Mammalian Protein Extraction Reagent (Thermo Scientific, Rockford, IL, USA) supplemented with HALT Protease Inhibitor Cocktail (Thermo Scientific) and Phosphatase Inhibitor Cocktail (Thermo Scientific). Fifteen micrograms of total protein were electrophoresed per well on a SDS-polyacrylamide gel and transferred onto Immobilon-FL PVDF membranes (Millipore, Billerica, MA, USA). Membranes were then incubated with primary antibodies (Additional file 6: Table S4) and probed with a mixture of IRDye polyclonal secondary antibodies (LI-COR Biosciences, Lincoln, NE, USA). Images were read with an Odyssey infrared imaging system (LI-COR Biosciences) and the average density of each band was measured with ImageJ software (National Institutes of Health, Bethesda, MD, USA).

### Enzyme-linked immunosorbent assay (ELISA)

Control and CCR7 shRNA MDA-MB-231 cells were cultured in serum-free media and treated with or without human CCL21/6Ckine (350 ng/ml, R&D Systems, Minneapolis, MN, USA) for 24 hours before supernatants were collected. Then, VEGF-C concentration in conditioned media was measured by ELISA (Quantikine Human VEGF-C Immunoassay, R&D Systems). For signaling studies, cells were pretreated with various concentrations of PI3 kinase inhibitor (LY294002) (Cell Signalling Technology, Danvers, MA, USA), AKT inhibitor (Akti-1/2) (Abcam, Cambridge, MA, USA), and MEK 1/2 inhibitor (U0126) (Cell Signalling). Tumor cells were then treated with CCL21/6Ckine for 24 hours and the level of VEGF-C secretion was determined. In order to quantify CCL21 protein secretion, MDA-MB-231 and HMVEC-dLy cells were maintained in 2D and 2D-matrix culture conditions and subjected to Quantikine Human 6Ckine Immunoassay (R&D Systems).

### Functional assays

#### Proliferation

Serum-starved HMVEC-dLy cells were seeded onto 96-well tissue-culture microplates, treated with various concentrations of CCL21/6Ckine (0, 100, 200, and 350 ng/ml) for 24 hours, and a cell proliferation ELISA BrdU (colorimetric) assay (Roche Applied Science, Indianapolis, IN, USA) was performed.

#### Migration

Cellular migration was assessed with Boyden chambers using Transwell® inserts (Corning Life Sciences, Oneonta, NY, USA) separated by a polycarbonate membrane with 8 μm pore opening placed within 24-well plates. A two hundred microliter suspension of serum-starved HMVEC-dLy cells at a concentration of 2×10^5^/ml were seeded in the upper chamber while various concentrations of CCL21/6Ckine (0, 100, 200, and 350 ng/ml) were added to serum-free media in the lower chamber. The assembled chambers were then incubated for 24 hours. After incubation, the cells from the top of the membrane were wiped off with cotton swabs whereas the migrated cells (from the bottom of the membrane) were fixed with cold methanol, stained with eosin/thiazine, and washed with distilled water. The membranes were then dried, cut with surgical blade, and fixed with mounting medium on a glass slide. Direct microscopic counting at 40 × magnification (Leica DFC 295, Leica Microsystems, Germany) of cells that have migrated to the lower side of the membrane was performed and a mean value for each sample was calculated.

#### Tube formation

HMVEC-dLy grown up in T75 flasks to near confluence (80%) were trypsinized and resuspended in endothelial basal media (EBM) (without any growth supplements) to a final concentration of 2×10^5^ cells/ml. Growth Factor Reduced Matrigel (BD Biosciences, Mississauga, ON, Canada) was thawed overnight at 4°C, diluted with cold EBM (1:1 dilution) and then a volume of 300 μl Matrigel solution/well was placed in six-well plates to solidify for 2 hours. After that, HMVEC-dLy cells (2 ml of cell suspension) were seeded on the solidified Matrigel under various concentrations of CCL21/6Ckine (0, 100, 200, and 350 ng/ml) for 24 hours. Tube formation was examined on an inverted microscope (100× magnification) at different time intervals. Images were randomly taken with Leica EC3 camera (Leica Microsystems) in different areas of the wells by selecting fields of view that were distinct and distant enough to not overlap with each other. The total length of the interconnected cells forming tubular structures was measured with ImageJ.

### Directed *in vivo* lymphangiogenesis assay

To study the loss of function, the highly aggressive MDA-MB-231 breast cancer cell line that expresses high endogenous levels of VEGF-C was used after CCR7 knockdown. To study the gain-of-function, the poorly aggressive MCF-7 breast cancer cell line that expresses low endogenous VEGF-C was used after CCL21 knockin. Four angioreactors (Trevigen, Gaithersburg, MD, USA) with identical conditions/mouse (4×10^4^ cells/angioreactor) were implanted into the dorsal flank of 6-to 8-week-old female nude mice (4 mice/condition) (Hsd.Athymic Nude-*Foxn1*^*nu*^*/Foxn1*^+^, Indianapolis, IN, USA) and the assay was carried out as previously described [43]. Three different approaches were employed for this purpose:

#### Spectrofluorimetry

To quantify lymphatic ingrowths, angioreactors were removed from surrounding tissues and cellular contents were retrieved. The fluorescence signal of Lyve-1, Prox-1, and CD31 markers (Additional file 6: Table S4) was measured with a FLUOstar Omega (Fisher Scientific, San Jose, CA, USA) spectrofluorimeter (excitation 584 nm, emission 620 nm).

#### Quantitative real-time PCR

To assess the expression of lymphangiogenic markers in the cellular contents of angioreactors, total mRNA was extracted and quantitative PCR was performed with specific murine probes (Additional file 5: Table S3).

#### Immunofluorescence staining

It was carried out in serial frozen sections of angioreactors as previously described [34,43] using appropriate antibodies (Additional file 6: Table S4). Representative images were taken with a LSM 510 META confocal microscope (Carl Zeiss Microscopy, Jena, Germany) and micro (blood) and lymphatic vessel densities (MVD/LVD) were assessed in dual immunostained sections as reported in the past [34].

### Statistical analysis

Statistical calculations were performed using GraphPad Prism software version 5 (GraphPad Software, La Jolla, CA, USA). All parametric data were analyzed with one-way ANOVA followed by Tukey-Kramer or Dunnett post-hoc comparisons. Student’s *t*-test was used when comparing two datasets and Pearson’s coefficient was employed to assess statistical correlations. Statistically relevant differences between means were accepted at p < 0.05.

## Additional files

Additional file 1: Figure S1. (A) Western blot and (B) real-time PCR analysis of CCR7, CCL21, and VEGF-C expression in T47D, MCF-7, Hs578t, and MDA-MB-231 breast cancer cell lines. GAPDH was used as an internal control. (C) CCL21 protein secretion by MDA-MB-231 as measured by ELISA in 2D and 2D-matrix conditions. Data are represented as mean ± SD (n = 3). (*) indicates statistical significant differences (p < 0.005). Additional file 2: Figure S2. (A) Immunofluorescence staining depicts the expression of CCR7. Green (Alexa fluor labeling) represents CCR7 protein expression and blue (DAPI) represents nuclei. Images were taken under 40× magnification. (B) Quantitative PCR of CCR7 mRNA expression in LECs obtained from angioreactors subject to different treatment conditions. Data is presented relative to growth factor-reduced basement membrane extract (BME) alone. Data are represented as mean ± SD (n = 3). (*) indicates statistical significant differences (p < 0.005). (C) Immunofluorescence staining of the lymphangiogenic marker Prox-1 showing its nuclear localization in LECs. Image was taken under 40× magnification. Additional file 3: Table S1. Demographic details and tumor characterization. Additional file 4: Table S2. Primers information for real-time PCR. Additional file 5: Table S3. Primer probe information for quantitative real-time PCR. Additional file 6: Table S4. Antibodies for Western blot, immunofluorescence and immunohistochemical analyses.

## Abbreviations

ACTB: Actin, beta
ANOVA: Analysis of variance
CCL21/6Ckine: C-C chemokine ligand 21
CCR7: C-C chemokine receptor 7
CD31: Cluster of differentiation 31
COX-2: Cyclooxygenase 2
DAPI: 4′, 6-diamidino-2-phenylindole
DIVLA: Directed *in vivo* lymphangiogenic assay
DNA: Deoxyribonucleic acid
ELISA: Enzyme-linked immunosorbent assay
EP1: EP4, Prostanoid receptor 1, 4
ERK1/2: Extracellular regulated kinase 1/2
GAPDH: Glyceraldehyde 3-phosphate dehydrogenase
HMVEC-dLyAd: Primary adult human dermal lymphatic microvascular endothelial cells
LVD: Lymphatic vessel densities
LYVE-1: Lymphatic vessel endothelial receptor-1
LECs: Lymphatic endothelial cells
MVD: Micro vessel densities
PI3K: Phosphatidylinositol 3-kinase
PROX-1: Prospero homebox protein-1
PKB/AKT: Protein kinase B
PGE2: Prostaglandin E2
RT-PCR: Reverse transcriptase polymerase chain reaction
VEGF-C: Vascular endothelial growth factor-C
VEGFR-3: Vascular endothelial growth factor receptor-3
