# Supplementary figures and images for: The role of CCL21/CCR7 chemokine axis in breast cancer-induced lymphangiogenesis
Source: Mol Cancer. 2015 Feb 10;14:35. doi: 10.1186/s12943-015-0306-4 (PMC4339430; doi:10.1186/s12943-015-0306-4)

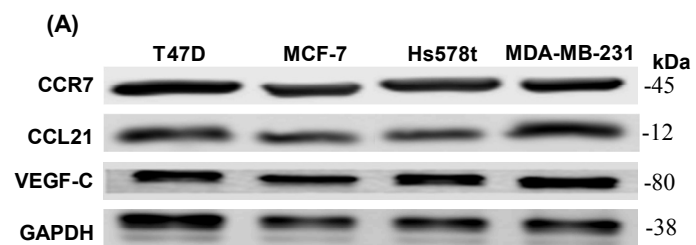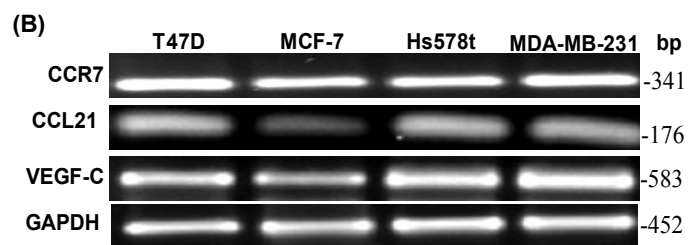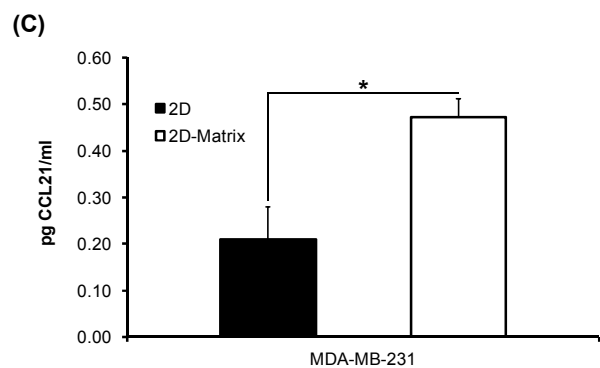

Supplement: Additional file 1: Figure S1. — (A) Western blot and (B) real-time PCR analysis of CCR7, CCL21, and VEGF-C expression in T47D, MCF-7, Hs578t, and MDA-MB-231 breast cancer cell lines. GAPDH was used as an internal control. (C) CCL21 protein secretion by MDA-MB-231 as measured by ELISA in 2D and 2D-matrix conditions. Data are represented as mean ± SD (n = 3). (*) indicates statistical significant differences (p < 0.005). [file 12943_2015_306_MOESM1_ESM.pdf]

(A)

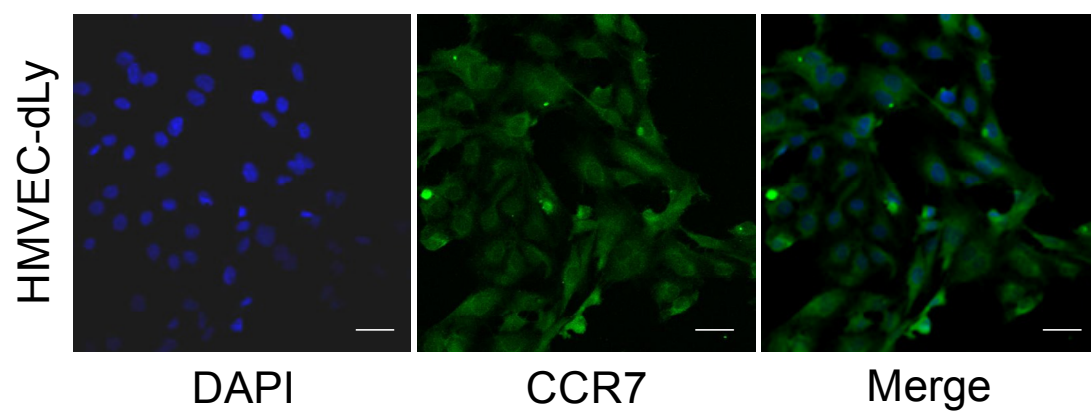

(B)

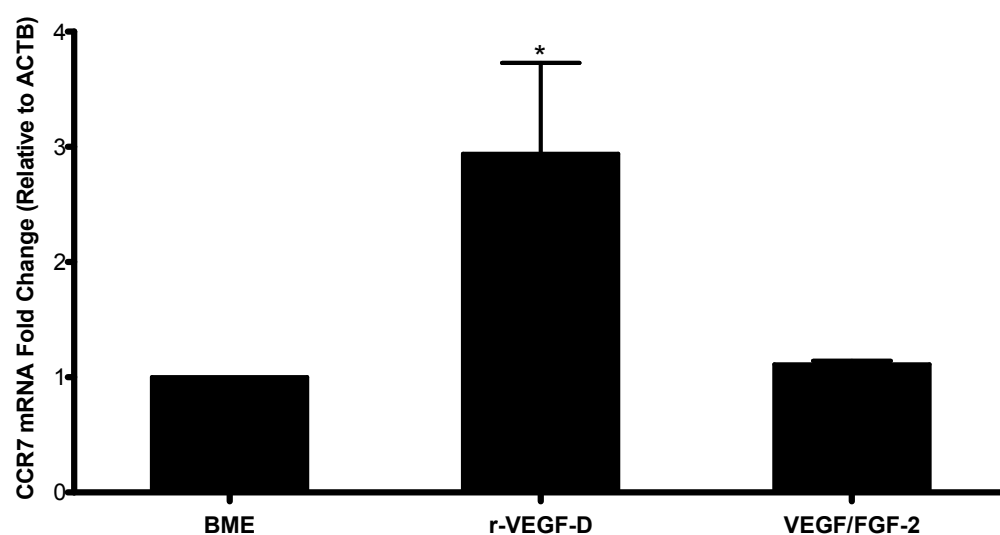

(C)

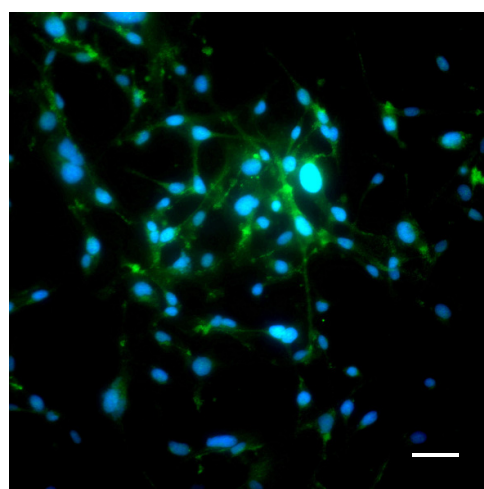

Supplement: Additional file 2: Figure S2. — (A) Immunofluorescence staining depicts the expression of CCR7. Green (Alexa fluor labeling) represents CCR7 protein expression and blue (DAPI) represents nuclei. Images were taken under 40× magnification. (B) Quantitative PCR of CCR7 mRNA expression in LECs obtained from angioreactors subject to different treatment conditions. Data is presented relative to growth factor-reduced basement membrane extract (BME) alone. Data are represented as mean ± SD (n = 3). (*) indicates statistical significant differences (p < 0.005). (C) Immunofluorescence staining of the lymphangiogenic marker Prox-1 showing its nuclear localization in LECs. Image was taken under 40× magnification. [file 12943_2015_306_MOESM2_ESM.pdf]
