# Supplementary material for: The role of CCL21/CCR7 chemokine axis in breast cancer-induced lymphangiogenesis
Source: Mol Cancer. 2015 Feb 10;14:35. doi: 10.1186/s12943-015-0306-4 (PMC4339430; doi:10.1186/s12943-015-0306-4)
Supplement: Additional file 3: Table S1. — Demographic details and tumor characterization. [file 12943_2015_306_MOESM3_ESM.docx]

**Additional file 3: Table S1: Demographic details and tumor characterization**

| **Subjects** | | **Controls**  **N= 20 (%)** | **Cancer**  **N= 105 (%)** |
| --- | --- | --- | --- |
| Sex | Male | 0 | 3 (2.8) |
|  | Female | 20 (100) | 102 (97.2) |
| Age distribution (years) | Range | 52 – 87 | 27 – 92 |
| Age (years) | Mean ± SD | 66 ± 11 | 64 ± 12 |
| Estrogen receptor (ER) status | Positive | N/A | 80 (76) |
|  | Negative | N/A | 19 (18) |
| Progesteron receptor (PR) status | Positive | N/A | 66 (62.9) |
|  | Negative | N/A | 33 (31) |
| HER2 status | Positive | N/A | 21 (20) |
|  | Negative | N/A | 68 (64.8) |
| ER, PR, HER2 status | Negative | N/A | 10 (9.5) |

| **Tumor Grade** | **N (%)** |
| --- | --- |
| I (low – well differentiated) | 7 (6.7) |
| II (intermediate – moderately differentiated) | 26 (24.76) |
| III (high – poorly differentiated) | 63 (60) |
| X (Unknown) | 9 (8.57) |
