# Supplementary material for: The role of CCL21/CCR7 chemokine axis in breast cancer-induced lymphangiogenesis
Source: Mol Cancer. 2015 Feb 10;14:35. doi: 10.1186/s12943-015-0306-4 (PMC4339430; doi:10.1186/s12943-015-0306-4)
Supplement: Additional file 4: Table S2. — Primers information for real-time PCR. [file 12943_2015_306_MOESM4_ESM.docx]

**Additional file 4: Table S2. Primers information for real-time PCR**

| **Primer** | **Sequence and Size** |
| --- | --- |
| CCR7 | forward 5′-GACCGATACCTACCTGCTCAACC-3′  reverse 5′-GCTCACTGCTGCTCCTCTGG-3′, 341 bp |
| CCL21 | forward 5'-CGCAGCTACCGGAAGCAG-3'  reverse 5'-CTGCCTGAGAGCGCTTGC-3',176 bp |
| VEGF-C | forward 5’- CGGGAGGTGTGTATAGATGTG-3  reverse 3’- ATTGGCTGGGGAAGAGTTTG-5’, 583bp |
| GAPDH | forward 5’-ACCACAGTCCATGCCATCAC-3’  reverse 3’- TCCACCACCCTGTTGCTGTA-5’,452 bp |
