# Supplementary material for: The role of CCL21/CCR7 chemokine axis in breast cancer-induced lymphangiogenesis
Source: Mol Cancer. 2015 Feb 10;14:35. doi: 10.1186/s12943-015-0306-4 (PMC4339430; doi:10.1186/s12943-015-0306-4)
Supplement: Additional file 5: Table S3. — Primer probe information for quantitative real-time PCR. [file 12943_2015_306_MOESM5_ESM.docx]

**Additional file 5: Table S3. Primer probe information
for quantitative real-time PCR**

| **Target Primer** | **Probe ID** |
| --- | --- |
| CCR7 | Hs99999080 |
| CCL21 | Hs99999110 |
| CCL19 | Hs00355524 |
| VEGF-C | Hs00153458 |
| LYVE-1 | Hs00272659 |
| Podoplanin | Hs00366761 |
| CD31 | Hs01065281 |
| GAPDH | Hs0275891 |
| ACTB | Hs99999903 |
| Lyve1 | Mm00475056_m1 |
| CD31 | Mm01242584_m1 |
| ACTB | 4352933E |
