# Supplementary material for: The role of CCL21/CCR7 chemokine axis in breast cancer-induced lymphangiogenesis
Source: Mol Cancer. 2015 Feb 10;14:35. doi: 10.1186/s12943-015-0306-4 (PMC4339430; doi:10.1186/s12943-015-0306-4)
Supplement: Additional file 6: Table S4. — Antibodies for Western blot, immunofluorescence and immunohistochemical analyses. [file 12943_2015_306_MOESM6_ESM.docx]

**Additional file 6: Table S4. Antibodies for Western blot,**

**immunofluorescence and immunohistochemical analyses**

| **Antibody** | **Concentration** | **Manufacturer** |
| --- | --- | --- |
| Monoclonal rabbit anti-CKR7/CCR7 (N-term) | 1:10000 (WB) | Epitomics |
| Monoclonal mouse  anti-human CCR7 | (Neutralization) | R&D Systems |
| Polyclonal goat  anti-human 6 Ckine | 1:200 (WB) | R&D Systems |
| Polyclonal rabbit anti-VEGFR3 | (Neutralization) | Bioss |
| Polyclonal goat  anti-VEGF-C (C-20) | 1:250 (WB) | Santa Cruz Biotechnology |
| Monoclonal rabbit anti-phospho-AKT (Ser 473) | 1:500 (WB) | Cell Signalling Technology |
| Monoclonal mouse  anti-AKT (pan) | 1:500 (WB) | Cell Signalling Technology |
| Polyclonal rabbit anti-phospho p44/42 MAPK (ERK 1/2 ) | 1:500 (WB) | Cell Signalling Technology |
| Polyclonal rabbit anti-p44/42 MAPK (ERK 1/2 ) | 1:500 (WB) | Cell Signalling Technology |
| Polyclonal rabbit  anti-mouse Lyve1 | 1:500 (IHC,IF) | AngioBio |
| Polyclonal rabbit  anti-mouse PROX-1 | 1:200 (IHC,IF) | Acris  Antibodies |
| Polyclonal rabbit  anti-mouse Podoplanin | 1:200 (IHC,IF) | Bioss |
| Monoclonal rat  anti-mouse CD31 | 1:200 (IHC,IF) | Santa Cruz  Biotechnology |
